# Validated Cohesive Zone Models for Epoxy-Based Adhesive Joints Between Steel and CFRP Composites for Multimaterial Structural Design in Transportation Applications

**DOI:** 10.3390/polym18030309

**Published:** 2026-01-23

**Authors:** Stanislav Špirk, Tomáš Kalina

**Affiliations:** Faculty of Mechanical Engineering, University of West Bohemia, 301 00 Pilsen, Czech Republic; tkalina@fst.zcu.cz

**Keywords:** epoxy adhesive, cohesive zone model, pam-crash, steel-cfrp joint, hybrid structure, transportation safety

## Abstract

This study presents the development, calibration, and validation of cohesive zone models (CZMs) for epoxy-based adhesive joints connecting stainless steel and CFRP composites. The objective of this study is to develop and rigorously validate cohesive zone models for epoxy-based adhesive joints between stainless steel and CFRP composites, ensuring their reliability for numerical simulations of structural failure under quasi-static and large-deformation conditions. The work focuses on accurately describing the quasi-static behaviour and failure mechanisms of hybrid adhesive interfaces, which are crucial for multimaterial structures in modern transportation systems. Experimental tests in Mode I (DCB) and Mode II (ENF) configurations were performed to determine the cohesive parameters of the structural adhesive SikaPower 1277. The obtained data were further analysed through analytical formulations and validated numerically using PAM-CRASH. Excellent agreement was achieved between experiments, analytical predictions, and simulations, confirming the reliability of the proposed material definitions under large deformations. The validated models were subsequently implemented in a large-scale numerical simulation of a bus rollover according to UN/ECE Regulation No. 66, demonstrating their applicability to real structural components. The results show that the developed cohesive zone models enable accurate prediction of failure initiation and propagation in adhesive joints between dissimilar materials. These findings provide a robust foundation for the design of lightweight, crashworthy structures in transportation and open new perspectives for integrating epoxy-based adhesives into additively manufactured hybrid metal–composite systems.

## 1. Introduction

The increasing demand for lightweight yet high-strength structures in the transportation industry has led to the extensive use of hybrid components that combine carbon-fiber-reinforced polymers (CFRP) with metallic materials such as steels and aluminum alloys [1,2,3]. Adhesive bonding represents one of the most efficient joining techniques for these multimaterial assemblies, providing uniform stress distribution and avoiding galvanic corrosion typical for mechanical fastening [4,5]. However, the reliable prediction of adhesive joint behavior remains a challenge due to the complex nonlinearities arising from the viscoelasticity of polymeric adhesives, local damage initiation, and mixed-mode fracture propagation [6,7].

To address these challenges, cohesive zone modeling (CZM) has become a powerful numerical approach capable of representing damage initiation and progressive delamination at interfaces between dissimilar materials [1,8]. CZM enables the simulation of both Mode I (opening) and Mode II (shear) fracture modes, as well as mixed-mode interactions, through the definition of traction–separation laws calibrated by experimental tests such as the Double-Cantilever Beam (DCB) and End-Notched Flexure (ENF) configurations [4,8,9]. Recent studies have shown that properly calibrated CZM models can accurately capture the fracture energy of epoxy-based adhesives and predict delamination behavior in CFRP/steel hybrid structures under quasi-static and impact conditions [2,3,8].

Recent studies have highlighted the increasing importance of adhesive bonding and co-curing technologies in composite and hybrid structures, particularly for transportation applications where lightweight design, structural integrity, and crashworthiness are critical. Experimental investigations have shown that joint configuration, fibre orientation, and manufacturing techniques significantly influence the mechanical response and failure behaviour of adhesively bonded composite joints. For instance, recent work has addressed the mechanical performance of unidirectional fibre-reinforced epoxy joints manufactured by adhesive bonding and co-curing techniques, demonstrating the strong dependence of joint behaviour on the selected joining approach and material architecture [10]. Similarly, advanced lamination strategies in co-cured CFRP joints have been shown to enhance shear and vibration performance, underlining the need for accurate modelling of adhesive interfaces in structurally demanding applications [11]. However, most existing studies remain focused on coupon-level testing or simplified representations of the adhesive layer, with limited emphasis on validated cohesive modelling approaches suitable for large-deformation or full-scale structural simulations.

Despite these advances, there remains a lack of experimentally calibrated cohesive zone models that are systematically validated across experimental, analytical, and numerical levels and subsequently applied to full-scale structural simulations relevant to transportation safety. In particular, the transferability of cohesive parameters from standard fracture tests to large-scale crash or rollover simulations has not been sufficiently addressed. The present study aims to bridge this gap by combining Mode I and Mode II experimental characterisation with analytical formulations and explicit numerical simulations, culminating in a full-scale bus rollover analysis. While the present work focuses on conventional manufacturing routes, the validated modelling framework provides a basis for future extensions towards hybrid and additively manufactured structural components.

The present work focuses on the validation of cohesive zone models for epoxy-based adhesive joints used in multimaterial structural designs for transportation applications. Experimental characterization through DCB and ENF tests was performed to determine the cohesive parameters of SikaPower 1277 epoxy adhesive, followed by numerical and analytical simulations aimed at reproducing real joint behavior under pure Mode I and Mode II loadings. Finally, the validated material models were implemented into the explicit finite element code Visual Crash-PAM 18.0 to evaluate their performance in a full-scale rollover simulation according to UNECE Regulation No. 66, representing a typical crash-relevant loading condition for bus superstructures. The findings contribute to the broader goal of developing reliable adhesive models for future 3D-printed lightweight hybrid components for lightweight vehicles [5,9].

## 2. Materials and Methods

### 2.1. Material Testing of Stainless Steel 1.4003

Stainless steel grade 1.4003 (W.Nr., DIN: X2CrNi12) is a ferritic stainless steel with a nominal chemical composition of C < 0.03%, Cr 10.5–12.5%, and Ni 0.3–1.0%. The material was supplied in two different forms—cold-rolled sheets and rectangular hollow sections (RHS)—in several thicknesses relevant to the structural components of the bus body framework. To account for the potential differences in mechanical properties between these product forms, uniaxial tensile tests were performed on specimens extracted from both sheets and RHS profiles. All tensile tests were conducted according to the ČSN EN ISO 6892-1 standard under quasi-static loading conditions [12].

**Figure 1:**
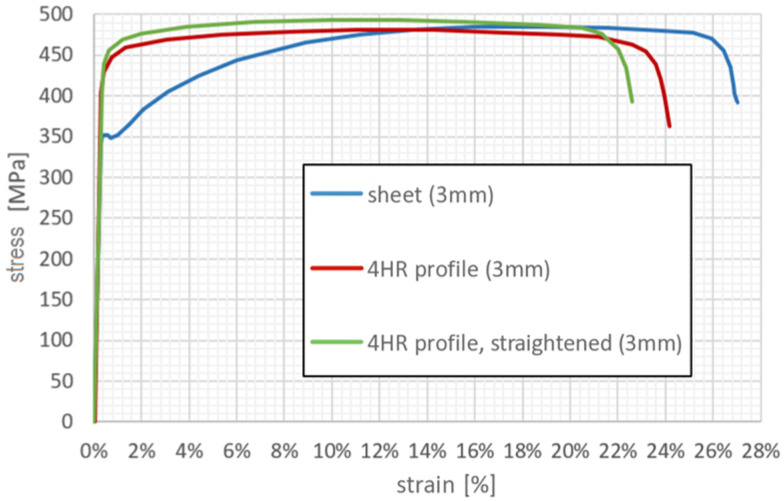
Representative engineering stress–strain curves for 3 mm stainless-steel sheet and RHS specimens.

**Figure 2:**
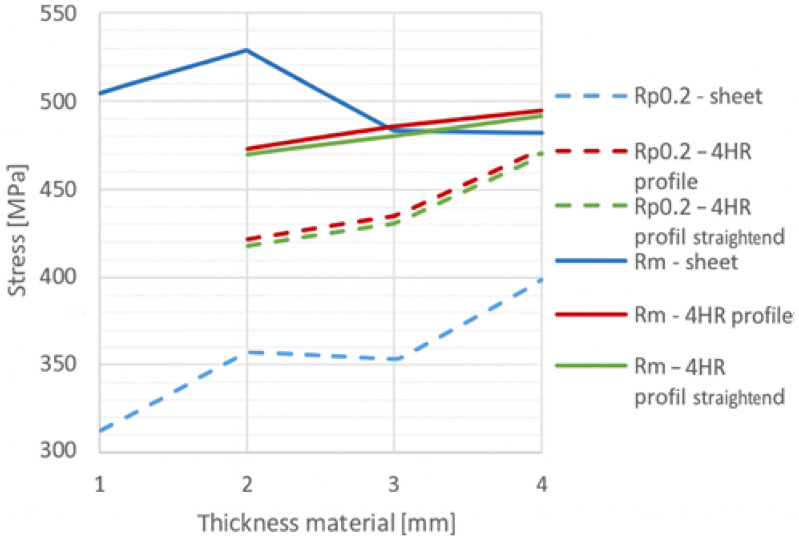
Comparison of yield and ultimate tensile strengths for various thicknesses and product origins.

Representative engineering stress–strain curves for 3 mm thick sheet and RHS specimens are shown in Figure 1. The results reveal that sheet specimens exhibit a significantly lower yield strength compared to RHS samples, while their ultimate tensile strengths are nearly identical. This behaviour was consistent across all tested thicknesses and product origins. A summary comparison of the yield and ultimate tensile strengths for the different specimen types and thicknesses is presented in Figure 2. Here, $R_{p0.2}$ denotes the 0.2% proof stress (yield strength), $R_{m}$ represents the ultimate tensile strength, and 4HR refers to the rectangular hollow section specimens with a wall thickness of 4 mm.

### 2.2. Material Testing of CFRP SIKA Carbodur S812

**Figure 3:**
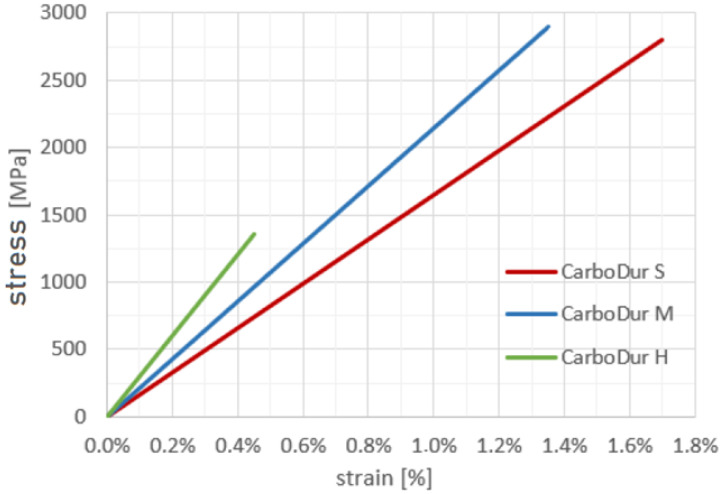
Comparison of tensile stress–strain responses of three SIKA Carbodur CFRP laminates (types S, M, and H). The Carbodur S laminate shows a modulus of elasticity most comparable to steel, making it suitable for hybrid reinforcement applications.

The SIKA Carbodur laminates are unidirectional carbon-fibre-reinforced polymer (CFRP) strips designed for the strengthening of concrete, timber, masonry, steel, and fibre-reinforced structures. The laminates are produced by embedding high-strength carbon fibres into an epoxy matrix, followed by curing at an elevated temperature of 180 °C. This manufacturing process ensures a uniform fibre alignment, high longitudinal stiffness, and excellent adhesion to structural substrates when bonded with compatible epoxy adhesives. The unidirectional nature of the laminate provides superior tensile strength in the fibre direction, making it suitable for use as an external reinforcement element in hybrid steel–composite systems. Among the available SIKA Carbodur laminates, type Carbodur S was selected for the experimental program and numerical modelling (see Figure 3). This laminate exhibits a longitudinal elastic modulus of approximately 170 GPa, which is the closest to that of stainless steel 1.4003, thus ensuring compatible deformation behaviour and efficient stress transfer in the hybrid steel–CFRP structure. The Carbodur S laminate also provides sufficient tensile strength and strain capacity while maintaining good handling characteristics during bonding and curing.

### 2.3. Material Testing of Adhesive SikaPower 1277

**Figure 4:**
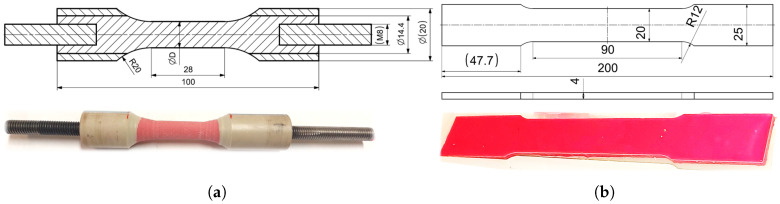
(**a**) Cylindrical specimens of SikaPower 1277 adhesive prepared in polymer tubes. (**b**) Flat specimens cast in silicone moulds for tensile testing.

The adhesive SikaPower 1277 is a high-strength and impact-resistant two-component structural epoxy adhesive that cures at room temperature. It is suitable for bonding steel, aluminium alloys, and composite laminates such as CFRP and GFRP, which are widely used in the transportation industry. To determine its mechanical properties in tension, both cylindrical and flat specimens of the cured adhesive were prepared. The cylindrical specimens were produced in two different diameters by injecting the adhesive into polymer tubes, which were subsequently machined after curing, as illustrated in Figure 4a. The flat specimens were cast into silicone moulds, as shown in Figure 4b. The mechanical characterisation of the epoxy-based adhesive SikaPower 1277 was performed following relevant international standards for structural adhesives, including tensile and shear testing procedures, as specified in the manufacturer’s technical documentation and applicable ISO and ASTM standards.

**Figure 5:**
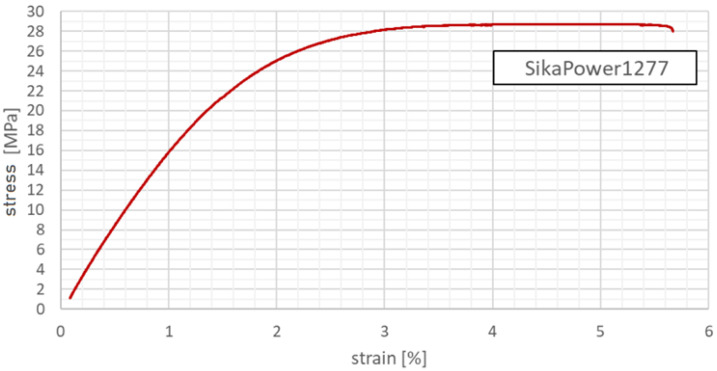
Tensile test example results of specimen of SikaPower 1277 adhesive.

The tensile properties (Figure 5) obtained from all three specimen geometries were found to be very similar, confirming the consistency of the curing process and the isotropic behaviour of the adhesive in tension. In addition to tensile tests, shear properties of the SikaPower 1277 adhesive were evaluated using several types of lap-shear specimens designed to represent different joint configurations.

### 2.4. Determination of Mode I Parameters—DCB Test

**Figure 6:**
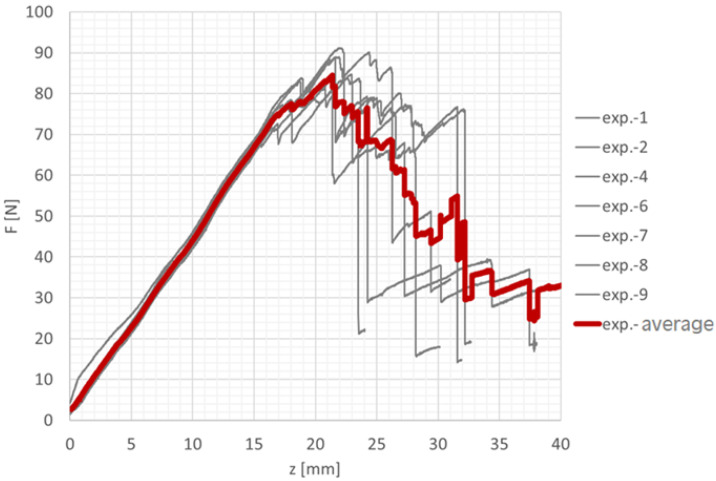
Experimental load–displacement curves obtained from DCB tests.

The parameters for Mode I were determined using the Double-Cantilever Beam (DCB) test, which involves a specimen with a pre-crack loaded in a direction normal to the adhesive layer, causing opening of the crack front [13]. In accordance with ASTM D5528 standard [14], the SikaPower 1277 was used as the adhesive, the machined steel blocks served as hinges, and both adherends were carbon laminates manufactured from GG200t prepreg. The experimental tests were performed on a Zwick/Roell Z050 testing machine with a maximum load capacity of 50 kN. The specimens were loaded under displacement control at a rate of 5 mm/min. The force–displacement response was recorded using a load cell and an extensometer attached to the upper grip (Figure 6). The DCB test procedure was conducted in accordance with ASTM D5528, including specimen geometry, initial pre-crack length, and loading configuration.

The maximum load before the first onset of failure was in the range of

(1) $$F_{n,\max}=\langle 78÷91\rangle \;N,$$

with the averaged curve reaching

(2) $$F_{n,\max,\mathrm{avg}}=84.5\;N.$$

The normal stiffness of the adhesive layer with a thickness h=0.5mm was calculated according to Equation (3):

(3) $$k_{03}=\frac{E_{n}}{h},$$

where $k_{03}$ is the normal stiffness of the adhesive, $E_{n}$ is the tensile modulus, and *h* is the adhesive layer thickness.

For the given thickness, the stiffness was determined as

(4) $$k_{03}\left(h=0.5\right)=\langle 3076÷4000\rangle \;{N/\mathrm{mm}}^{3}.$$

The fracture energy $G_{n}$ for Mode I was determined using the analytical expression derived from Euler–Bernoulli beam theory:

(5) $$G_{n}=\frac{F_{n,\max}^{2}a^{2}}{bE_{x,B}J_{y,B}}=\frac{12F_{n,\max}^{2}a^{2}}{b^{2}h^{3}E_{x,B}}.$$

Alternatively, the strain energy release rate can be expressed as a function of the crack tip opening displacement $\delta_{n}$:

(6) $$G_{n}=\frac{3F_{n,\max}\;\delta_{n}}{2ab}.$$

where

(7) $$\delta_{n}=\frac{2F_{n,\max}a^{3}}{3E_{x,B}J_{y,B}}+\frac{F_{n,\max}h^{2}a}{4G_{xz,B}J_{y,B}}.$$

Here, $F_{n,\max}$ is the maximum opening force before adhesive failure, while *a* and *b* are defined geometric parameters [15]. The analytical representation of the DCB test is possible. Based on the determined fracture energy $G_{n}$ and the known geometric and material properties of the bonded beams, simplified analytical relationships can be derived to describe the load–displacement behaviour of the DCB test. The following expressions (Equations (8) and (9)) define the force response in the elastic and damage phases of the test:

(8) $$F_{\mathrm{elast}}\left(z\right)=\frac{3E_{x,B}J_{y,B}}{2a^{3}}\;z,$$

(9) $$F_{\mathrm{failure}}\left(z\right)=\frac{b^{3/4}{\left(E_{x,B}J_{y,B}\right)}^{1/4}}{\sqrt{3}}\;G_{n}^{3/4}\;\frac{1}{\sqrt{z}},$$

where *z* is the specimen opening displacement (i.e., the actuator displacement or hinge separation), $F_{\mathrm{elast}}\left(z\right)$ represents the force response in the elastic region, and $F_{\mathrm{failure}}\left(z\right)$ corresponds to the cohesive failure phase of the adhesive.

The relationships constructed according to these analytical equations are illustrated in next figures. For parameters defined as ranges (such as $E_{x,B}$ and $G_{n}$), mean values were used for the plotted dependencies. A numerical model of the DCB test was developed in Visual Crash PAM 18.0 using the explicit VPS Solver 2022.0 (ESI Group). The bonded beams were modelled using underintegrated quadrilateral shell elements with an element size of 2.5 mm and five integration points through the thickness. This type of element was selected to ensure consistency with the modelling of larger structural assemblies using similar joint configurations. Under-integrated elements are approximately four times more computationally efficient than fully integrated ones, although they require monitoring of the artificial (hour-glass) energy to prevent non-physical deformation modes. The beams were assigned an elastic–plastic material model representing the carbon laminate adherends.

**Figure 7:**
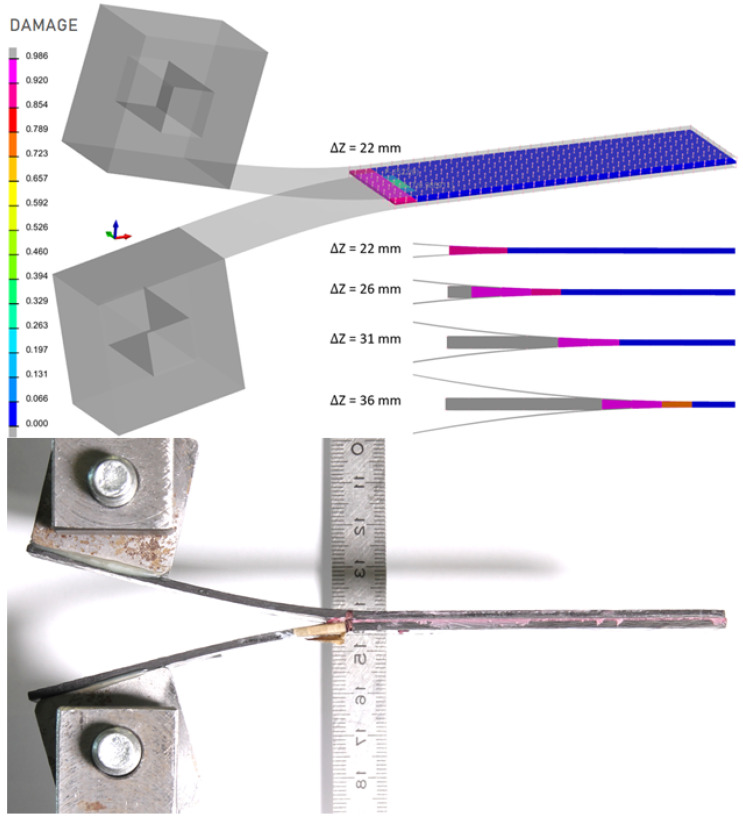
Numerical model of the DCB test created in Visual Crash PAM 18.0 and physical experiment.

The adhesive layer was modelled using COS3D cohesive elements of dimensions 2.5 × 2.5 × 0.5 mm Figure 7. Due to their specific formulation, the small element height does not limit the stable time step, unlike conventional 3D solid elements. The selected cohesive element size of 2.5 × 2.5 × 0.5 mm was chosen as a compromise between numerical accuracy and computational efficiency. The in-plane dimensions were selected to ensure sufficient resolution of the damage process zone along the adhesive layer, while the thickness corresponds to the nominal adhesive layer thickness. The mesh density was verified to provide stable and mesh-independent global responses, in line with recommendations reported in the literature for cohesive zone modelling of adhesive joints. The adherends were connected to the cohesive layer using TIED contacts, ensuring full kinematic compatibility between the surfaces. The rigid loading blocks were modelled as rigid bodies, as their deformation influence is negligible.

The mid-surface spacing of the shell elements was defined as half the beam thickness on each side of the adhesive layer, preserving the correct joint geometry and stiffness representation. The model was loaded by applying a synchronized prescribed displacement at the centres of both rigid blocks in the opposite directions. Rotations about the Y-axis were allowed to simulate hinge behaviour.

The loading rate used in the experiment (5 mm/min) was not feasible for explicit simulation; therefore, the prescribed displacement was linearly ramped within a simulation time of 5000 ms and 500 ms. No significant differences in material response were observed between these two loading durations, and the strain rate remained below approximately 5.897 × 10^−4^ ms^−1^

A series of simulations was performed to calibrate the cohesive parameters of the adhesive layer within the experimentally determined ranges, aiming to achieve the best correlation between the numerical and experimental force–displacement responses.

### 2.5. Determination of Mode II Parameters—ENF Test

**Figure 8:**
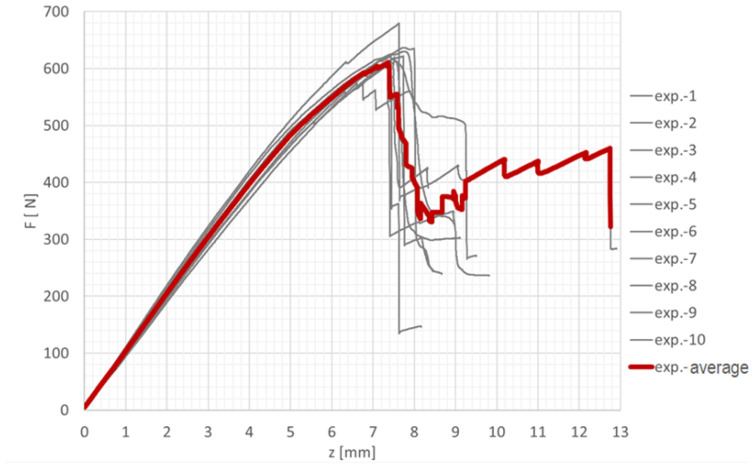
Graphical representation of the measured data from the ENF test.

The parameters for Mode II were determined using the End-Notched Flexure (ENF) test, which consists of a specimen with an artificially introduced crack supported on two beams and loaded at midspan, similar to a three-point bending test. This configuration produces shear stresses at the crack tip. In accordance with the ASTM D7905 standard [16], ENF specimens were designed using the adhesive SikaPower 1277, while both bonded beams were made of carbon laminate prepared from GG200t prepreg. The experimental testing was conducted on a Zwick/Roell Z050 testing machine with a maximum load capacity of 50 kN. The specimens were loaded under displacement control at a rate of 0.5 mm/min. The force–displacement response was recorded using a load cell and an extensometer attached to the upper fixture. Figure 8 shows the recorded curves from individual specimens as well as their average curve. The maximum load before the first failure occurred ranged from 570 to 680 N, while the peak of the averaged curve reached approximately 620 N.

The shear stiffness of the adhesive layer with a thickness of 0.9 mm was determined using previously established material properties of the adhesive according to Equation (10). The resulting shear stiffness was calculated as

(10) $$k_{01}\left(h=0.9\;\mathrm{mm}\right)=\langle 656÷855\rangle \;N/{\mathrm{mm}}^{3}.$$

The relationship used for this calculation is given by

(11) $$k_{01}=\frac{E_{S}}{h},$$

where $k_{01}$ is the shear stiffness of the adhesive, $E_{S}$ is the shear modulus (the notation $E_{S}$ was chosen instead of the conventional symbol *G* to avoid confusion with the fracture energy symbol), and *h* is the adhesive layer thickness. The shear modulus $E_{S}$ was determined from the tensile modulus of the adhesive using Equation (12):

(12) $$E_{S}=\frac{E_{n}}{2(1+\nu )},$$

where ν is the Poisson ratio. The resulting values of $E_{S}$ were in the range of 590–770 MPa.

The fracture energy $G_{s}$ for Mode II was determined analytically according to the Euler–Bernoulli beam theory, resulting in values ranging from 1979 to 2816N/m. The analytical expression used is given in Equation (13):

(13) $$G_{s}=\frac{9F_{s,\max}^{2}a^{2}}{16\;b^{2}h^{3}E_{x,B}}=\frac{9F_{s,\max}^{2}a^{2}\;\delta }{2b\left(3a^{3}+2L^{3}\right)},$$

where $\delta_{n}$, the deflection at maximum load, was determined according to Equation (14):

(14) $$\delta_{n}=\frac{F_{s,\max}\left(3a^{3}+2L^{3}\right)}{8E_{x,B}bh^{3}}.$$

In these relations, $F_{s,\max}$ is the maximum load before adhesive failure, and *a*, *b*, and *L* are geometric parameters of the specimen. The terms $E_{x,B}$, $J_{y,B}$, and $G_{xz,B}$ refer to the elastic and shear properties of the bonded beams and are defined according to their geometrical and material characteristics.

Based on the determined fracture energy $G_{s}$ and the known geometric and material characteristics of the bonded beams, a simplified analytical representation of the ENF test can be constructed using Equations (15) and (16). The elastic and failure parts of the response can be described by:

(15) $$z_{\mathrm{elast}}=\frac{F}{k_{B}}=\frac{12a^{3}+L^{3}}{384E_{x,B}J_{y,B}}\;F_{\mathrm{elast}}\left(z\right),$$

(16) $$z_{\mathrm{failure}}=\frac{F_{\mathrm{failure}}\;L^{3}}{384E_{x,B}J_{y,B}}+\frac{16}{F_{\mathrm{failure}}^{2}}\;\sqrt{E_{x,B}J_{y,B}}{\left(\frac{b\;G_{s}}{3}\right)}^{3/2}.$$

Here, *z* represents the specimen deflection, $F_{\mathrm{elast}}\left(z\right)$ is the force in the elastic region, and $F_{\mathrm{failure}}$ is the force response during the cohesive failure phase.

**Figure 9:**
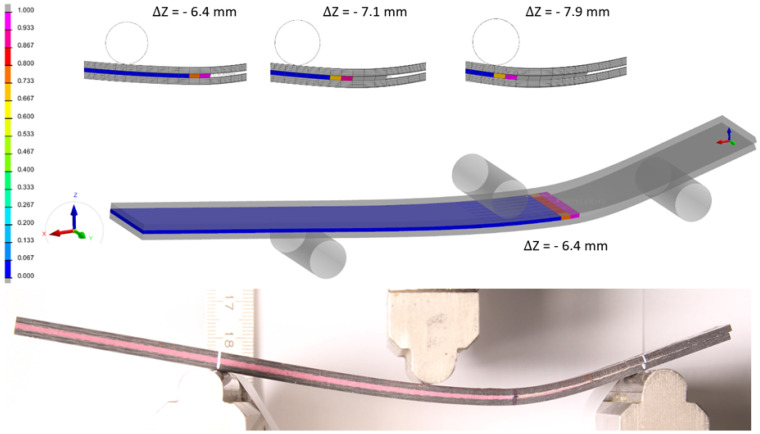
Numerical model of the ENF test created in Visual Crash-PAM 18.0 and physical experiment.

The analytical curves constructed according to these relations are shown in Figure 9 in next paragraph. For parameters defined as ranges of measured values, such as $E_{x,B}$ and $G_{s}$, mean values were used for the plotted dependencies. A numerical model of the ENF test was developed in Visual Crash-PAM 18.0 using the explicit VPS Solver 2022.0. The model geometry and material properties were identical to those used for the DCB test, with the only difference being the adhesive layer thickness of 0.9 mm. The bonded beams and adhesive layer were modelled using the same element types as in the DCB model. The beams were supported by two perfectly rigid cylindrical bodies that were fully constrained in all degrees of freedom (1, 1, 1, 1, 1, 1). Loading was applied using a third rigid cylindrical body positioned at midspan above the specimen, with displacement prescribed in the negative Z-direction. The center of this loading cylinder was guided in the Z-axis (1, 1, 0, 1, 1, 1), and a vertical displacement of −15 mm was imposed.

For the numerical simulation, the prescribed displacement was applied linearly over durations of 5000 ms and later 500 ms. No visible differences were observed between the two loading durations, indicating that the strain rate remained relatively low, on the order of 5.897 × 10^−4^ ms, corresponding to quasi-static loading. Because the beams were modelled as mid-surfaces of shell elements, the actual thickness of the laminate was accounted for by offsetting the mid-surfaces from the adhesive layer by half of the beam thickness. If the mid-surfaces were instead coincident with the upper and lower faces of the adhesive layer, the model stiffness would be significantly underestimated. The total height of the beam section has a substantial effect on its bending stiffness, as the second moment of area of a rectangular section increases with the cube of its height. Therefore, even small deviations in the adhesive–beam interface position can noticeably influence the overall stiffness of the specimen.

The beams and adhesive layer were connected using TIED contacts, ensuring full kinematic compatibility between the shell and cohesive elements. Contact type 33 with a friction coefficient of 0.1 was defined between the specimen and all three cylinders. Additionally, a self-contact type 36 was defined between the unbonded beam ends, allowing them to touch when the adhesive layer was fully deformed. The contact distance was set equal to the total adhesive thickness, and a friction coefficient of 0.1 was also applied.

A series of numerical simulations of the ENF specimen was performed, varying the input parameters of the cohesive elements representing the adhesive layer within the experimentally determined ranges. The main calibrated parameters were Es (or k01), Gs, and Rs, max, el, which strongly influence the shear behaviour of the cohesive layer.

### 2.6. Experiment for Validation of Material Model Parameters

**Figure 10:**
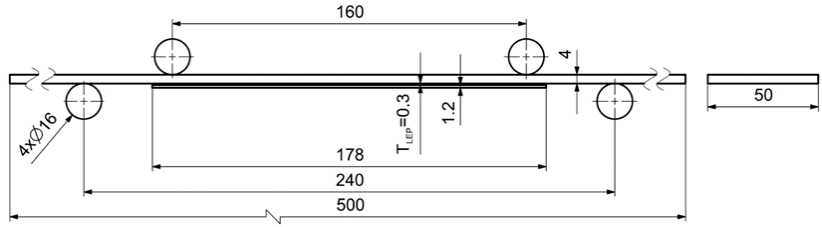
Geometry of the designed specimen for the four-point bending test of a steel plate reinforced with a CarboDur S512 strip.

Based on the previous experimental measurements, analytical relations, and numerical simulations, the cohesive parameters describing the adhesive SikaPower 1277 were determined and subsequently validated. The validation was carried out on a four-point bending test of a stainless steel plate (grade 1.4003) reinforced with a unidirectional CFRP laminate Sika CarboDur S512. The geometry of the specimen is shown in Figure 10.

**Figure 11:**
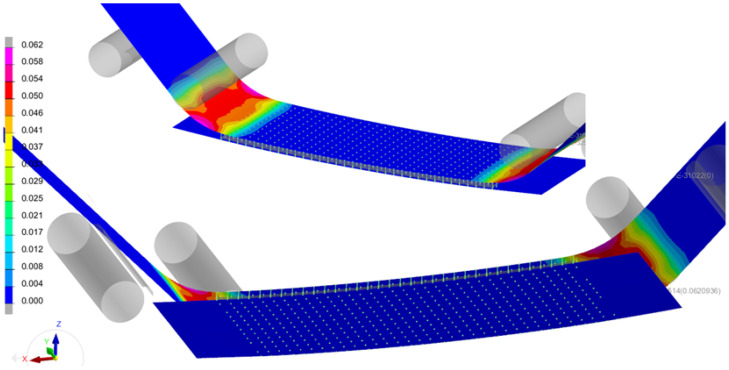
Visualization of the strain field distribution [–] in the steel and CFRP at a loading displacement of 30 mm.

**Figure 12:**
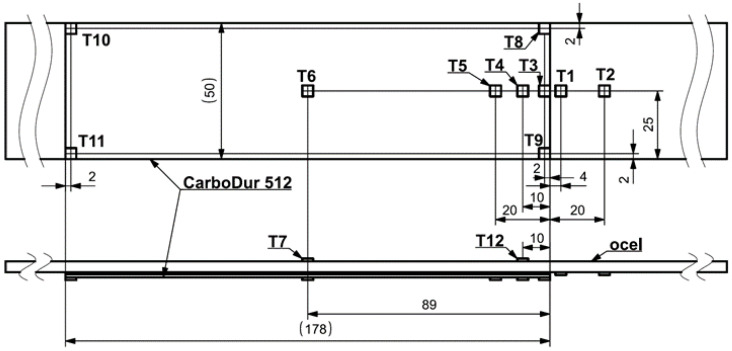
Visualization of the strain gauge positions on the steel plate specimen reinforced with a CarboDur S512 strip.

A numerical model of the test was developed in Visual Crash-PAM 18.0 (Figure 11), including the contact regions and friction conditions observed during the physical experiment. The beam was loaded by a synchronized vertical displacement of −30 mm applied through two upper rigid cylinders. Contact with a friction coefficient of 0.1 was defined between all cylinders and the specimen. For the purpose of the experimental measurement, the specimen was equipped with several strain gauges, the positions of which are shown in Figure 12.

The specimen was instrumented with strain gauges to capture the deformation field for validation purposes. Both the simulation and the experiment showed plastic deformation of the steel plate below the loading cylinders and cohesive failure of the adhesive layer in the same region [17]. The experimentally observed failure length of the adhesive ranged between 9 and 14 mm, while the numerical model predicted 15 mm, which is in very good agreement considering the 3 mm element size. This test involved combined shear and normal loading, as confirmed by the fracture energy components Gn and Gs, which maintained an approximate ratio of 1:2.4 throughout the cohesive failure process.

## 3. Results

The primary outcome of this research is the development, calibration, and validation of material models for the steel, CFRP, and adhesive components used in hybrid adhesive joints. These models were constructed based on the experimental tests, analytical derivations, and numerical simulations presented in the previous sections. The results of all validation procedures confirmed that the implemented models accurately capture both the elastic–plastic behaviour and the failure characteristics of the studied materials. The following subsections provide detailed descriptions of the final validated models, which can be directly implemented in Visual Crash-PAM 18.0 for reliable simulation of adhesive joints with similar materials and geometries [18].

### 3.1. Final Validated Material Models

Three numerical material models were developed based on the experimental tests described in the previous sections. The models represent the individual constituents of the hybrid steel–composite structure. For specific load cases, the models were validated by performing comprehensive mechanical tests and comparing the experimental results with numerical simulations. This validation confirmed the accuracy of the calibrated parameters in reproducing the stiffness, strength, and failure modes of the tested specimens. All parameters are presented in a form directly compatible with the PAM-CRASH solver, and the style of the tables follows the input data structure used in PAM-CRASH keyword format. This ensures that the models can be easily implemented and reused for further numerical analyses of hybrid bus structures and related components. The base structural material used for the steel profiles is a commercially available ferritic stainless steel 1.4003, commonly applied in bus and coach body frameworks. The material model was defined for rectangular hollow sections with a wall thickness of approximately 3 mm, corresponding to the dimensions used in the physical prototypes. The constitutive behaviour of the steel was represented using the MAT 105—“Elastic Plastic ITR with ISO Damage” formulation in PAM-CRASH. Plastic deformation was described by a simple Krupkowski powerlaw hardening model, which allows straightforward adjustment of the stress–strain curve for various grades of steel. Material failure was interpreted as the descending part of the stress–strain curve observed in tensile testing, corresponding to the onset of necking. Final element deletion was controlled either by the REL-THIN parameter, defining the relative shell-thickness reduction, or by the maximum plastic strain limit (EPSIpmax), which in this case corresponds to the compressive failure condition. The resulting parameter set (Table 1) provides a robust and numerically stable description of the elastic–plastic response of thin-walled stainless-steel members under tensile and compressive loading.

The carbon-fibre-reinforced polymer (CFRP) reinforcement was represented by a unidirectional lamella SIKA Carbodur S812, characterised by high stiffness in the fibre direction and brittle failure behaviour. Since the reinforcement is a unidirectional composite, the numerical model was significantly simplified and implemented as MAT 103—Elastic Plastic Iterative Hill in PAM-CRASH. Several alternative modelling approaches were tested, including fully orthotropic definitions and simplified models with negligible bending stiffness (pure tension along fibres). However, the most stable and physically reasonable solution was obtained using a simplified isotropic formulation, which proved to be a valid idealization for this application. Although the CFRP exhibits an elastic modulus comparable to steel—ensuring good mechanical compatibility—the material has a very high yield stress and almost no plastic deformation beyond the linear range. Failure occurs at approximately 5% strain due to brittle fracture, which was incorporated into the model through a low plastic strain limit (EPSIpmax = 0.05). The final parameter set (Table 2) thus provides an effective yet computationally efficient representation of the CFRP lamella behaviour in hybrid steel–composite structures.

The structural adhesive SikaPower 1277, recommended by the manufacturer for bonding CFRP laminates to stainless-steel substrates, was implemented using the MAT 305—COS3D cohesive zone model in PAM-CRASH. This formulation describes the traction–separation law for three-dimensional cohesive elements and allows for an independent definition of the normal (Mode I) and shear (Mode II) responses. The parameters summarized in Table 3 define both the static and dynamic fracture characteristics, enabling a mixed-mode interaction governed by the ETA exponent [19]. The PSLOPE coefficient controls the stiffness degradation rate for both fracture modes, while En specifies the normal stiffness of the adhesive layer. The adhesive was modelled with a single layer of three-dimensional solid elements, where the element thickness corresponds to the actual adhesive layer thickness (typically around 0.1 mm). In explicit simulations, such small physical thickness would normally reduce the stable time step dramatically; however, the COS3D cohesive formulation internally compensates for this effect, allowing the time step to remain unaffected. Although the iterative scheme of the COS3D material model increases the computational time roughly fourfold, this is still considerably more efficient than a solution based on conventional thin solid elements. The cohesive elements were connected to the surrounding structural parts using TIED contacts defined by material model 371 (Slink Elink Tied Kinematic). The setup ensures full kinematic continuity between the adhesive and the adherends. The only significant parameter in this interface definition is IDABEN = 2, which specifies that the normal vector of the cohesive element is updated according to the rotation of the master node. The adopted configuration proved numerically stable and accurately reproduced the experimental stiffness and failure modes of the adhesive joints under both tensile and shear loading conditions [20].

### 3.2. Agreement Between Experimental, Numerical, and Analytical Results for Cohesive Failure in Mode I

**Figure 13:**
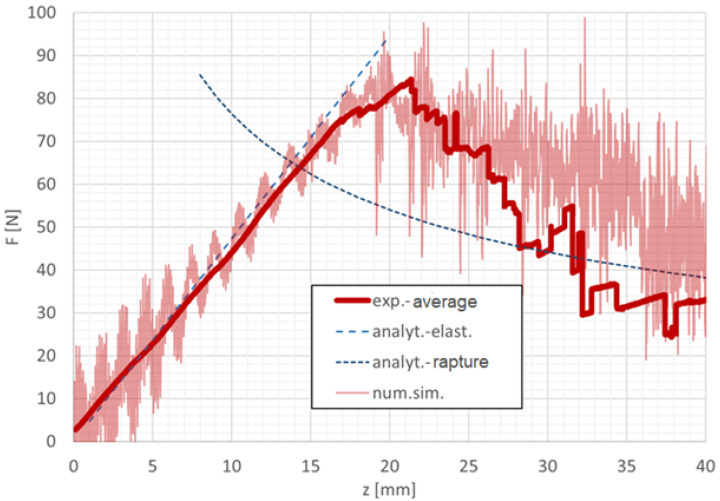
Graphical comparison of the averaged experimental data, numerical simulation, and analytical solution of the DCB test.

At the crack tip prior to the onset of failure, the normal strain in the cohesive layer was recorded as $\varepsilon_{zz}=0.2307$, corresponding to an opening displacement of $\delta_{n}=115\;$µm. This value is in very good agreement with both the numerical simulation and the analytical solution of the DCB test, as illustrated in Figure 13, where the average experimental curve is compared with the numerical and analytical results.

**Figure 14:**
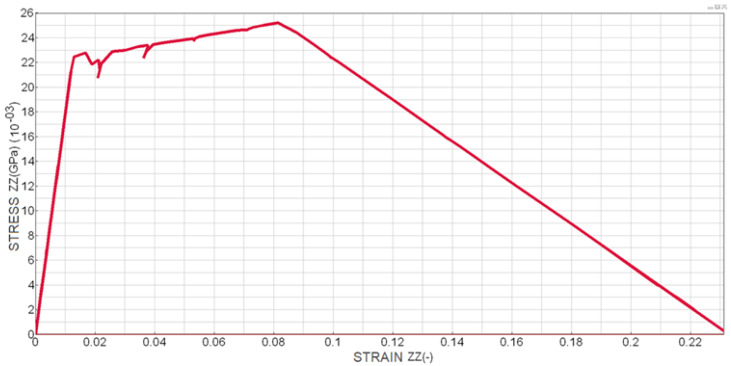
Normal stress–strain relationship in the ZZ direction for elements at the crack tip during the DCB test.

The distribution of normal stress versus normal strain in the cohesive zone (Figure 14) confirms the expected cohesive response and progressive degradation of stiffness prior to complete separation. In accordance with the theoretical assumptions, the deformation energy components indicate that the Mode I DCB test represents a purely normal loading state of the adhesive layer, with the normal component contributing more than 99.999% to the total deformation energy.

#### 3.2.1. Agreement Between Experimental, Numerical, and Analytical Results for Cohesive Failure in Mode II

**Figure 15:**
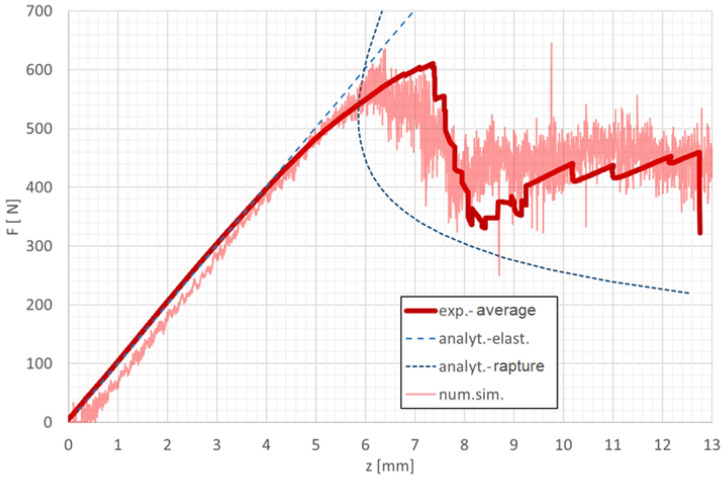
Graphical comparison of the averaged experimental data, numerical simulation, and analytical solution of the ENF test.

In accordance with the theoretical assumption that the ENF configuration represents a purely shear loading of the adhesive layer, the results confirm that the shear component of the deformation energy contributes more than 99.999% to the total deformation energy [21]. The comparison of the averaged experimental data, numerical simulation, and analytical solution of the ENF test is shown in Figure 15, demonstrating excellent agreement in the overall force–displacement response as well as in the onset and progression of failure [22].

**Figure 16:**
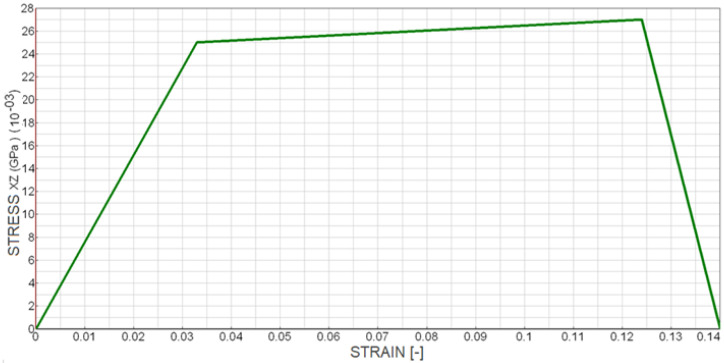
Shear stress–strain relationship in the XZ plane for elements at the crack tip during the ENF test.

The evolution of shear stress versus shear strain for the cohesive elements located at the crack tip, depicted in Figure 16, clearly illustrates the characteristic softening behaviour and stiffness degradation prior to complete debonding. These findings confirm that the validated cohesive zone model accurately reproduces the experimentally observed shear-dominated failure mechanism in Mode II loading. The post-peak softening behaviour observed in Figure 16 was captured using an explicit time integration scheme. Numerical stability during the softening phase was ensured by appropriate time step control and by employing a regularised cohesive zone formulation, which prevents localisation-induced numerical instabilities. This approach allowed stable simulation of damage initiation and progressive failure in the adhesive layer.

#### 3.2.2. Validation of Material Model Parameters

**Figure 17:**
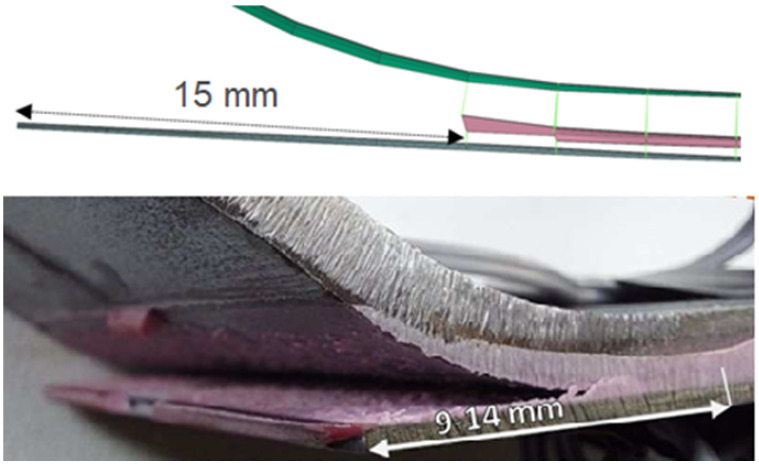
Sheet specimen with CarboDur S512 strip—comparison of numerical simulation and experimental results after unloading.

The results of both the numerical simulation and the experimental measurement show the onset of plastic deformation in the steel plate beneath the loading rollers, accompanied by cohesive failure of the adhesive layer in the same region. A comparison between the simulation and the experiment is presented in Figure 17, demonstrating very good agreement in the deformation of the steel plate. In the experimental specimen, the failure of the adhesive layer was identified over a length of approximately 9–14 mm, while the simulation predicted a failure length of 15 mm, which represents an excellent correlation considering that the characteristic element size in the model is approximately 3 mm.

**Figure 18:**
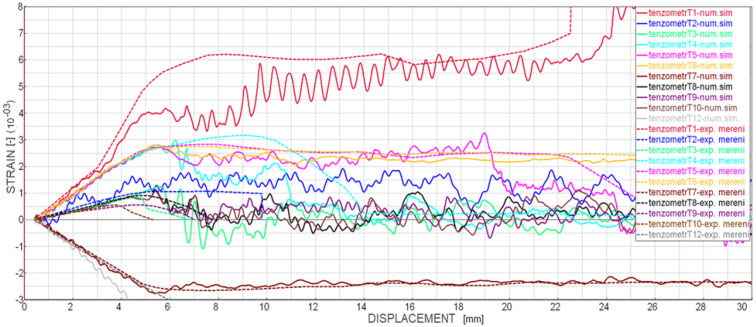
Comparison of longitudinal strain (XX) dependencies obtained from strain gauges and numerical simulation for the steel specimen with CarboDur S512 strip. The sudden drop in strain recorded by gauge No. 4 at a displacement of approximately 14 mm indicates the onset of laminate separation, which locally alters the load transfer within the joint.

To further validate the material model, the strain gauge readings obtained during the four-point bending test were compared with numerical predictions (Figure 18). Strain gauge No. 11 malfunctioned during the experiment and is therefore not displayed. However, the results from gauges No. 8, 9, 10, and 11 are theoretically expected to be identical. For the remaining gauges, a good agreement was observed between the numerical simulation and the experimental data. In the numerical simulation, the CFRP laminate exhibited oscillations caused by the significantly higher loading rate compared to the experiment, which resulted in minor fluctuations of strain values around zero even after the adhesive layer had completely detached. The highest strain values were, as expected, recorded by gauge T1, located on the bottom surface of the steel plate near the laminate front edge, where the largest plastic deformation of steel occurs due to the geometry of the test. Gauge No. 4, positioned within the laminate region that eventually undergoes full separation from the steel, shows a drop in strain at a roller displacement of approximately 14 mm. Gauges No. 6 and 7, situated near the midspan on the laminate and steel surfaces, respectively, display nearly constant strain throughout the test after the initial loading phase.

## 4. Discussion

The validated material models developed in this study demonstrated a very good correlation between experimental results, analytical predictions, and numerical simulations [23]. The cohesive zone formulations implemented in PAM-CRASH reproduced both the initiation and propagation of adhesive failure in Modes I and II with high accuracy. The validation experiments confirmed that the proposed models are capable of realistically describing the quasi-static behaviour of epoxy-based adhesive joints [15] under large deformations. Minor differences observed between the experimental and numerical results can be attributed mainly to the simplifications in the numerical representation of the adhesive layer and to the loading rate effects inherent to explicit solvers [24].

The overall comparison indicates that the cohesive parameters identified in laboratory-scale tests are transferable to larger-scale structural simulations without additional calibration, which demonstrates the robustness of the developed approach. The models thus provide a reliable foundation for predicting the mechanical response and failure behaviour of multimaterial adhesive joints in steel–CFRP hybrid structures [25].

**Figure 19:**
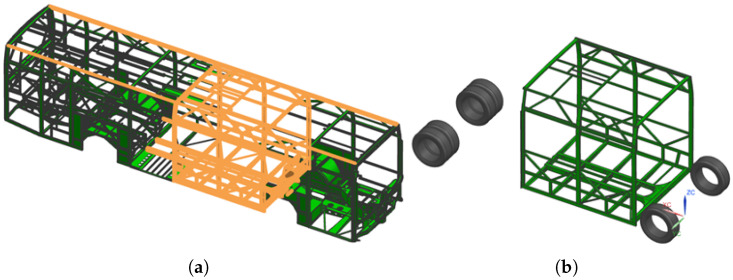
3D model of (**a**) the complete bus body and (**b**) the selected bus segment.

As a practical application, the validated models were used in a numerical rollover simulation according to the UN/ECE Regulation No. 66 for a bus body structure. Based on the source CAD model of the prototype vehicle by the manufacturer, a 3D model of a selected bus segment was created, comprising three of the nine vertical pillars of the body frame (Figure 19b), highlighted within the full bus geometry in Figure 19a. This segment was selected as the most compliant section of the structure, since the front and rear parts are stiffened by additional substructures such as wheel housings. In the absence of detailed seat models, the manufacturer specified equivalent reinforcements to represent their structural effect—four diagonal beams (60 × 40 × 2 mm rectangular hollow sections) placed between the floor and the sidewalls, shown in light brown in Figure 19a. The total mass of the steel structure was 494 kg, while the required overall segment mass of 3186 kg was achieved by introducing non-structural mass uniformly distributed among the model elements. The duration of the rollover event was preliminarily set to 1000 ms, with a calculated timestep of 5.897 × 10^−4^ ms.

**Figure 20:**
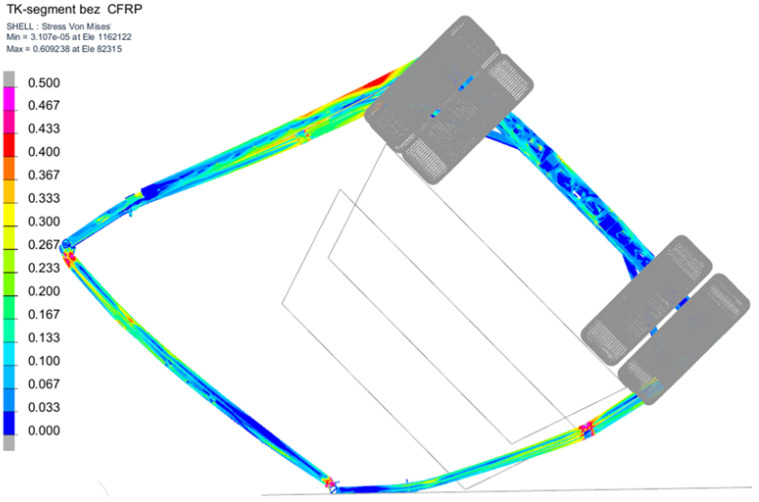
Segment at t = 270 ms showing maximum deformation and template intrusion; von Mises stress [GPa].

These simulations (Figure 20) will be followed by an experimental rollover test to further verify the predictive capability of the validated cohesive zone models under real structural loading conditions. Comparable validation procedures have been used for vehicle safety simulations, for instance in tram–pedestrian impact studies with complex material models [26,27].

## 5. Conclusions

This study presented the development and validation of cohesive zone models for epoxy-based adhesive joints between stainless steel and CFRP composites. The material parameters were derived from a series of mechanical tests, including DCB and ENF configurations, and verified through analytical formulations and numerical simulations. The validated models demonstrated excellent predictive capability under quasi-static loading with large deformations, showing close agreement with experimental observations. The bonding behavior observed under tensile loading is consistent with previous research on FRP-to-steel joints [28]. Although environmental effects are known to influence the long-term performance of adhesive joints, the present study focuses on quasi-static mechanical behaviour under controlled laboratory conditions. Environmental durability aspects, as discussed in the literature [29,30], are therefore considered outside the scope of this work and will be addressed in future studies.

The proposed models were successfully implemented in PAM-CRASH and applied to the simulation of a bus rollover according to UN/ECE Regulation No. 66. The results confirmed that the validated material definitions can be employed in large-scale structural analyses of transportation components, enabling accurate prediction of adhesive joint behaviour under combined loading conditions [31].

These findings contribute to the broader field of multimaterial design for transportation safety and sustainability. While the present work is limited to conventionally manufactured steel–CFRP joints, the presented modelling approach provides a solid foundation for future investigations of hybrid and additively manufactured structures, where reliable prediction of adhesive failure will remain a key challenge [31]. Such extensions represent a promising direction for the next generation of lightweight transportation systems.

## Figures and Tables

**Table 1:** Material model for stainless steel 1.4003 implemented in PAM-CRASH (MAT 105).

Keyword	Unit	Value	Description
RHO	kg/mm^3^	$7.85\times 10^{-6}$	Material density
ISINT	—	0	Shell integration rule
ISHG	—	4	Elements hourglass prevention
E	GPa	187	Elastic modulus
sigmOPTN	—	KRUPK	Yield option (plasticity model)
NU	—	0.28	Poisson’s ratio
k	GPa	0.75437	Plastic hardening parameter (strength coefficient)
EPSI0	—	0.0575	Strain at onset of hardening
n	—	0.194	Exponent of the power-law curve
SIGMAmax	GPa	0	Not used (no upper stress limit defined)
REL_THIN	—	0.72	Ratio of thickness/original thickness for element elimination
StrainOPT	—	Ini Strain	Strain options
EPSIi	—	0.3	Strain at damage initiation
EPSI1	—	0.5	Strain at damage initiation
d1	—	0.2	Intermediate damage parameter
EPSIu	—	0.6	Strain at failure completion
dU	—	0.6	Ultimate damage value
EPSIpmax	—	1.52	Strain limit for element elimination
EPMX	—	0.14	Keyword for selecting Generic user criterion output
KSI	—	0.1	Proportional damping

**Table 2:** Material model for CFRP SIKA Carbodur S812 implemented in PAM-CRASH (MAT 103).

Keyword	Unit	Value	Description
RHO	kg/mm^3^	$1.6\times 10^{-6}$	Material density
ISINT	—	0	Shell integration rule
ISHG	—	4	Elements hourglass prevention
E	GPa	165	Elastic modulus
sigmOPTN	—	Yield Stress	Yield option (plasticity model)
SIGMAy	GPa	3	Yield stress for single stress–strain curve
NU	—	0.26	Poisson’s ratio
E1	GPa	1	Plastic tangent modulus
SIGMA1	GPa	3.1	True stress (for E1)
EPSIpmax	—	0.05	Strain limit for element elimination
REL_THIN	—	0.92	Ratio of thickness/original thickness for element elimination
KSI	—	0.1	Proportional damping

**Table 3:** Material model for SikaPower 1277 implemented in PAM-CRASH (MAT 305).

Keyword	Unit	Value	Description
RHO	kg/mm^3^	$1.19\times 10^{-6}$	Material density
ISINT	—	0	Interface formulation type
ISHG	—	0	Hourglass control (off)
KSI	—	0.1	Stiffness-proportional damping ratio
IELAST	—	0	Elastic formulation flag
IDELA	—	1	Interaction method
ETA	—	6.79	Quasi-static loading mode criterion exponent
Ncycle	—	10	Number of cycles for stress reduction
NFEQD	—	10	Number of cycles for filtered displacements
IDRUP	—	0	Failure mode flag (off)
PSLOPE	—	0.0231	Hardening coefficient (both modes)
En	GPa	1.6	Normal stiffness (modulus)
SIGSn	MPa	0.0311	Max elastic stress (static mode)
R_SIGSn	—	$1\times 10^{-6}$	Strain rate for SIGSn
SIGDn	MPa	0.08083	Max elastic stress (dynamic mode)
R_SIGDn	—	10000	Strain rate for SIGDn
EFRSn	—	0.0039	Fracture energy (static mode)
R_EFRSn	—	$1\times 10^{-6}$	Strain rate for EFRSn
EFRDn	—	0.0072	Fracture energy (dynamic mode)
R_EFRDn	—	10000	Strain rate for EFRDn
PCn	N/mm	0.449	Normal plasticity coefficient
Es	GPa	0.37	Shear stiffness (modulus)
SIGSs	MPa	0.0231	Max elastic stress (static mode)
R_SIGSs	—	$1\times 10^{-6}$	Strain rate for SIGSs
SIGDs	MPa	0.04933	Max elastic stress (dynamic mode)
R_SIGDs	—	10000	Strain rate for SIGDs
EFRSs	—	0.0231	Fracture energy (static mode)
R_EFRSs	—	$1\times 10^{-6}$	Strain rate for EFRSs
EFRDs	—	0.02394	Fracture energy (dynamic mode)
R_EFRDs	—	10000	Strain rate for EFRDs
PCs	N/mm	0.49	Shear plasticity coefficient

## Data Availability

The experimental data used in this study—including raw DCB and ENF measurements, processed datasets, numerical model input files (VCPAM/VPS), and validation results—are openly available in the Zenodo Data Repository at the following permanent link: https://zenodo.org/records/18130892. The dataset is published under an open license in accordance with the Open Science requirements of the OP JAK project. A preprint of this manuscript is also available on Zenodo: https://zenodo.org/records/18200300.

## Abbreviations

The following abbreviations are used in this manuscript:

ASTM	American Society for Testing and Materials
CFRP	Carbon Fiber Reinforced Polymer
CZM	Cohesive Zone Model
DCB	Double Cantilever Beam (Mode I test)
ENF	End Notched Flexure (Mode II test)
ESI	Engineering Systems International (developer of Visual Crash-PAM)
FEA	Finite Element Analysis
